# A Real-Time Speech Separation Method Based on Camera and Microphone Array Sensors Fusion Approach

**DOI:** 10.3390/s20123527

**Published:** 2020-06-22

**Authors:** Ching-Feng Liu, Wei-Siang Ciou, Peng-Ting Chen, Yi-Chun Du

**Affiliations:** 1 Department of Electrical Engineering, Southern Taiwan University of Science and Technology, Tainan 71005, Taiwan; wtcen@hotmail.com (C.-F.L.); ma32d201@stust.edu.tw (W.-S.C.); 2 Department of Otolaryngology, Head and Neck Surgery, Chi Mei Medical Center, Tainan 71004, Taiwan; 3 Department of Biomedical Engineering & Medical Device Innovation Center, National Cheng Kung University, Tainan 70105, Taiwan; chen@bme.ncku.edu.tw or

**Keywords:** non-stationary, real-time speech separation method, microphone array, beamforming, asynchronous update

## Abstract

In the context of assisted human, identifying and enhancing non-stationary speech targets speech in various noise environments, such as a cocktail party, is an important issue for real-time speech separation. Previous studies mostly used microphone signal processing to perform target speech separation and analysis, such as feature recognition through a large amount of training data and supervised machine learning. The method was suitable for stationary noise suppression, but relatively limited for non-stationary noise and difficult to meet the real-time processing requirement. In this study, we propose a real-time speech separation method based on an approach that combines an optical camera and a microphone array. The method was divided into two stages. Stage 1 used computer vision technology with the camera to detect and identify interest targets and evaluate source angles and distance. Stage 2 used beamforming technology with microphone array to enhance and separate the target speech sound. The asynchronous update function was utilized to integrate the beamforming control and speech processing to reduce the effect of the processing delay. The experimental results show that the noise reduction in various stationary and non-stationary noise environments were 6.1 dB and 5.2 dB respectively. The response time of speech processing was less than 10ms, which meets the requirements of a real-time system. The proposed method has high potential to be applied in auxiliary listening systems or machine language processing like intelligent personal assistant.

## 1. Introduction

In recent years, the number of patients suffering from hearing loss has increased year by year. According to the World Health Organization, around 466 million people worldwide have disabling hearing loss, out of which 34 million are children. Annually, hearing loss costs USD750 billion worldwide. As the population grows and ages, it is estimated that 900 million people will suffer from hearing loss by 2050 [1]. Patients with hearing loss have difficulty in communicating with others, especially when they are in a multi-voice environment, which will impair speech comprehension and cause social isolation. Such medical condition not only causes declining life quality but also several mental conditions [2]. Traditionally, speech enhancement has been studied as a signal processing problem. Some recent methods have been based on supervised learning, including learning the discriminative patterns of speech, speakers, and background noise from training data. There have been good results for suppressing steady-state noise, such as feedback sound, wind sound, and white noise; however, these approaches are not ideal for speech enhancement from a specific target or real-time processing in a multiple human-sound environment using hearing aids. In a noisy environment, people with hearing loss need to improve their signal-to-noise ratio (SNR) more than the average person to have the same level of speech comprehension. Previous studies have shown that for every decibel increase in SNR, the hearing impaired can improve their comprehension of language by 10%. Therefore, from the results of the study, the enhancement of the target sound source is of greater importance [3,4]. The application of Computational Auditory Scene Analysis (CASA) to speech separation based on human perception on spectro-temporal properties of sounds to mimic human’s ability in hearing. Thus, the CASA usually used no more than two microphones, it is still difficult to achieve the requirement of real-time processing [5]. The other kind of speaker speech separation methods based on multiple microphones or microphone array, like independent component analysis (ICA), adaptive decorrelation filtering (ADF) and beamforming also do not perform well if only used single method under all situations, especially in various noise environment [6].

Previous studies have used beamforming technology to improve the performance of auxiliary listening systems or machine language processing [7,8]. In addition to the Time Difference of Arrival (TDOA) and frequency characteristics, space information can also be used to form beams according to the location of speech sources to improve speech enhancement efficiency. In this type of method, noise speech is divided into different frequency bands, and the gain corresponding to the specific frequency band is adjusted to enhance speech intelligibility according to the use environment and the degree of hearing loss of the patients [9,10,11,12]. Beamforming technology is based on the space filtering of a microphone array, typically by using a delay-and-sum beamformer (DSB) and the generalized side-lobe canceller (GSC) algorithm after speech data has been accepted by a microphone [13,14]. In 2017, Zohourian et al. studied the effectiveness of a microphone array with beamforming technology. They used four microphones distributed over binaural hearing aids and performed sound source localization for multiple azimuths based on beamforming technology and statistical model location algorithms. This method does not require binaural exploration training and the localization algorithms are integrated into a GSC to extract the desired speaker in the presence of competing speakers and background noise, as well as when the head of the listener turns. The experimental results show that in a noisy environment, the enhancement of signal sources was significantly effective [15]. In 2019, Picou et al. used unilateral and bilateral beamforming hearing aid products for clinical tests. The subjects wore commercially available unilateral and bilateral beamforming hearing aids to perform sentence identification tests in noisy environments with unilateral noise sources. The results show that the use of beamforming hearing aids is more effective for sentence recognition than omnidirectional products. Bilateral beamforming hearing aids had a better effect than unilateral beamforming hearing aids, and the overall data show that the noise location configurations in the experimental environment were not significantly related to the results of the unilateral and bilateral speech comprehension experiments [16]. However, as bilateral hearing aids correct all sound source signals through the same transition function, all sound sources will seem to from the same direction as the most energetic sound source and still cause confusion to users [17]. Currently, TDOA computation is performed on individual sound signals received through specific-angle microphone configurations, and beamforming methods implemented with GSC are widely utilized [18,19]. However, the main problem is that the adaptive function based on a specific-direction sound energy, when the noise source and the target sound source to converge closer and generate more energy than the target sound source, and this may cause the GSC to converge to the noise source. If there are a great deal of non-stationary human voices estimated under the GSC architecture, the target sound source will not be effectively enhanced. One of the important roles of beamforming technology is to correctly define and effectively enhance the source location of the target speech signal of interest (SOI).

In 2015, Erdogan et al. using deep recurrent neural networks to recognition-boosted speech enhancement. The experimental results showed that in experiments it yields uniformly better results in terms of SNR, which pointing to tighter integration of recognition with enhancement as a promising future direction [20]. In addition, in 2017, Feng et al. proposed a multimodal recurrent neural network (multimodal RNN) model to take into account the sequential characteristics of both audio and visual modalities for audio visual speech recognition. The experimental results showed the performance improvements comparing to the known highest audio-visual recognition, and confirm the robustness of our multimodal RNN model [21]. It is worth noting that in 2018, Ephrat et al. proposed a cocktail party effect solution to the SOI target selection problem, which was presented by the research unit of Google [22]. Their study proposed an integrated image recognition and speech analysis model for target sound source feature enhancement and background noise suppression through a large amount of training data and convolutional neural networks. The results showed that after thousands of hours of training, their system could separate and enhance target speech up to 16 dB. However, the method is offline and the system operation time is more than 3 s. Although such a structure can effectively separate and enhance human voices, the study did not specifically explore how to provide immediate speech enhancement and enhancement for the daily use of hearing aids. In 2019, to effectively resolve the target selection problem of SOI, Lin et al. proposed a set of hearing aid design schemas for the Field Programmable Gate Array (FPGA)-based and image processing technique. The experimental results indicated that the phonetic intelligibility of SOI can be improved by estimating TDOA through image processing and selecting the target location directly with an adaptive beamformer in a noisy environment. Moreover, they proposed an FPGA architecture based on video and speech processing, and analyzed its effectiveness using a backend host system. However, there is a lack of discussion on speech processing in real time, especially on how to synchronize video and speech processing. It is necessary to make a breakthrough in this aspect without affecting the immediate speech output [2,23].

Although the beamforming method has excellent performance and lower processing time, it needs speaker location and direction information [6]. The current study proposes a real-time speech enhancement and separation method. It utilizes an image identifying and tracking method to find and locate the target speaker and then calculate the angle and distance for beamforming to enhance the target sound source with an array microphone. In addition, Stone et al. conducted extensive studies on the tolerability of hearing aid latency, and the results indicated that the interference increases as the latency increases. For patients with mild to moderate hearing loss, latency of less than 10 ms can be considered as an interference phenomenon. Once the latency is more than 30 ms, users start to feel uncomfortable [24,25]. Therefore, in order to realize instantaneous non-stationary human voice processing in image processing, this study used asynchronous update technology to ensure real-time voice processing and output. Subsequently, the spectrum labeling function was used to enhance the target sound source.

## 2. Materials and Methods

### 2.1. System Architecture and Implementation

**Figure 1:**
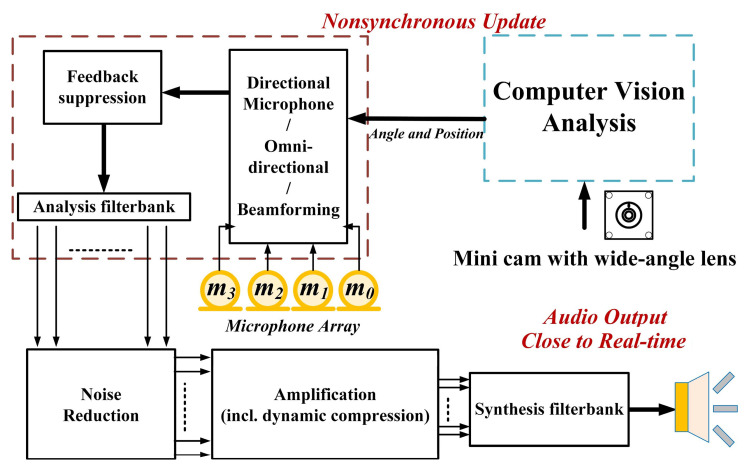
Signal processing of the proposed device.

A real-time adaptive method based on beamforming and computer vision (CV) technologies for speech enhancement was proposed in this study, and its architecture is shown in Figure 1. The system included a USB 2.0 cam with a 120-degree angle of view. Video was captured by the lens and processed with CV program through an H-Computing system in Hikey 970 platform. After identifying the target by CV and evaluating the angle to obtain a more precise TDOA for beamforming module. The interface system is based on PC used Microsoft C# to develop a real-time monitoring program for the display and peripheral hardware communication. Finally, the sound of a SOI which had been superposed and enhanced, could be output instantaneously over the earphone.

### 2.2. Computer Vision (CV) Analysis

In this study, commercial wide-angle Camera (OV2710 RER-USBFHD01, OmniVision, Santa Clara, CA, USA) was used as the image source of CV. These lenses provided the same 120 degrees of visual angle as the human eye for retrieving images of the user’s surroundings. The image sensor of the lens provided a high-resolution selection with a 2.1 mm focal length, 1080p 30 FPS and 720p 60 FPS, and a resolution of 1920 × 1080 pixels. First, an open source CV library was used to perform computer vision processing. In this study, the Haar classifier built into the OpenCV-based cv2 tool was used to detect the facial and ocular features of speech sources. Based on the measured eye size and position, the vertical distance and angle between the webcam and the speech target could be evaluated and the TDOA could be further calculated.

#### 2.2.1. Method of Evaluating TDOA by CV

In the proposed method, human face detection was obtained via a USB 2.0 webcam, the range was corrected to treat the face as a normalized basis based on human face feature points (mouth corner, nose, eyes) as anchor points, and the vertical and horizontal distances and angle of the target speech were further evaluated to calculate the TDOA value. After that, the focal length (FL) of the lens was 2.1 mm, the initial distance between the lens and SOI (DLS) was 160 cm, and the head circumference (HC) range were given as male: 13.1–15.9 cm and female: 12.5–15.0 cm [26]. The normalized values of the human face pixels (HFP) were obtained from Equation (1) and then we utilized it to update the distance and angle between the lens and SOI. According to the test, in the boundary conditions of male and female, the detection pixel gap was below 150, and there was no significant difference in the results of proposed CV detection. As the processing time needed for image recognition was still delayed, the asynchronous update method was used to process the image recognition in this study in order to meet the delay time of less than 10 ms in the use of hearing aids.

(1) $$\mathrm{HFP}=\frac{\mathrm{FL}\;\times \;\mathrm{HC}}{\mathrm{DLS}}$$

**Figure 2:**
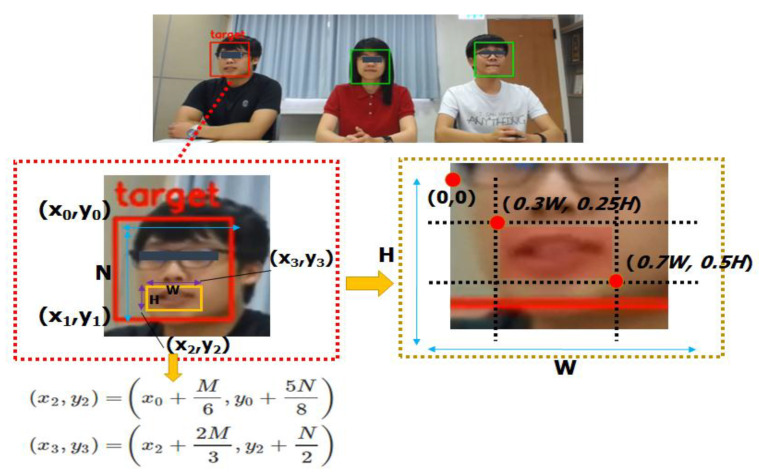
Redistribution of human face and lip features.

Based on the normalization of common Equation (1), the target distance could be deduced according to the pixel change. In order to increase the accuracy of target selection, the system regarded the maximum lip change as the highest probability at the time of conversation, and the range of facial features at this time would be reduced to the mouth profile, as shown in Figure 2, where (*x*_0_, *y*_0_) and (*x*_1_, *y*_1_) represent the detected origin of the face region frame and the position of the human face bottom, with the size of MxN. Then, according to the reasonable eye, nose and lip position ratio of the human face, the frame position of the lips, namely WxH, was preliminarily segmented. In order to get a more precise lip position in the next step, this study sets the threshold value to eliminate areas that might not belong to the lip profile. In this way, it was possible to define the reasonable range at between 0.3 W–0.7 W and 0.25 H–0.5 H. Then, we obtained the lip pixel changes during the conversation according to the pixels of Equation (1) and the distance change. However, parameter corrections were required when the target was not perpendicular to the center of the lens. In general, the method of calculating the distance between the lens and the non-vertical target object in multi-person conversations could be deduced from the pixel and distance based on lip changes when facing the target objects. Then, the difference between the vertical distance and the non-vertical target distance from the center of the lens could be obtained according to the relation of linearity. Finally, the vertical and horizontal distances of the target could be obtained according to the Pythagorean theorem. The formula derivation and weight relation assignment are discussed in detail in the related research proposed by Lin et al. in 2018 [23].

**Figure 3:**
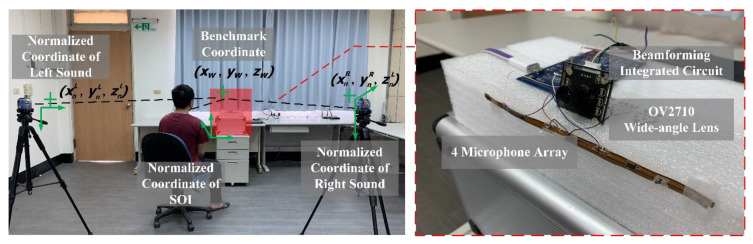
Three-dimensional positioning by using stereo triangulation. Virtual noise and SOI are needed to position arbitrary points in three dimensions.

Although the distance between the target and the lens could be effectively obtained by the pixel and distance conversion, the difference between the user and the target object was not only horizontal but also vertical. Therefore, beamforming corrections were made on the parameters through a three-dimensional vector, as shown in Figure 3.

The three-dimensional spatial location correction of beamforming could be performed through the difference in signal strength received by the array microphone. The single-microphone reception intensity *I* varies with the sound source in the normalized coordinate direction and is obtained through Equation (2), where *p*(*t*) is the intensity of the sound source and *V* is the particle velocity. Here, we set the *V* as 346 m/s based on the room temperature of 26 ℃ [27].

(2) $$I=\frac{1}{T}\int_{T}p\left(t\right)\;\times \;Vdt$$

Then, the sound intensity vector in three-dimensional space is composed of the sound intensity in three orthogonal directions (*x*, *y*, *z*), which can be obtained from Equation (3):

(3) $$\vec{I}=I_{x}{\vec{e}}_{x}+I_{y}{\vec{e}}_{y}+I_{z}{\vec{e}}_{z}$$

When the positions of the two normalized noise sources are known, the position of the target sound source can be corrected. Among them, the angle of the sound has been estimated and the two noise sources in the horizontal coordinates are known. Then, we can have:

(4) $$z_{w}{=z}_{n}-\;tan\varphi \sqrt{{{(x}_{w}\;-{\;x}_{n})}^{2}{+(y}_{w}\;-{\;y}_{n}{)}^{2}}\;$$

where, φ is a parameter estimate of the vertical angles. The problem now becomes how to estimate tan  φ. Detailed angle definition formulas can be obtained from the parameter correction method proposed by Chen et al. in the three-dimensional space positioning system [28].

#### 2.2.2. Asynchronous Update Function

**Figure 4:**
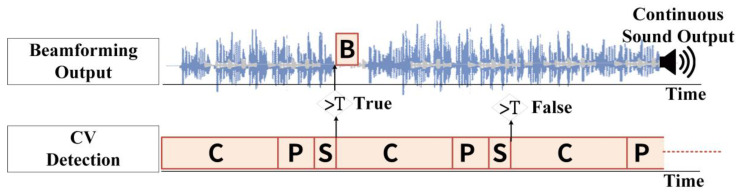
A parallel operation diagram of the asynchronous update function between CV and beamforming control.

With the development of automated visual technology and machine vision, multi-camera visual systems are becoming increasingly popular. However, in real-time visual analysis in visual sensor networks (VSNs), a large amount of computational power is required to perform image processing tasks within a specified time frame. When image data is synchronized to a central processing node for processing, a large amount of information at this time will result in a significant delay. By virtue of the nodal approach of distributed network processing, previous studies used asynchronous updates to increase the computational capacity available to the sensor nodes and reduce the system analysis time [29,30]. The parallel operation architecture for image processing using asynchronous update technology is shown in Figure 4.

In Figure 4, C denotes capturing image time (about 33 ms), P denotes image processing time (about 5 ms), S denotes angle and distance detection time (about 2 ms), and B denotes beamforming control delay time (about 7 ms). T denotes the threshold of last angle difference that for updating new beamforming control. We utilized the HiKey 970 platform for the CV processing with an embedded 3D graphics processor. An asynchronous update function was adopted the results from CV processing to update the parameters of beamforming control. The total CV processing time was about 40 ms (*C + P + S*), but the delay of beamforming control only 7 ± 0.3 ms. Under this structure, we can ensure that the output delay of the voice meets the requirements of the real-time system (<10 ms). Although it takes at least 40 ms to update the beamforming control due to image processing, in most cases, SOI does not move quickly in communication, and usually does not need to be updated constantly. In addition, this structure adds a threshold T in the Asynchronous update function, which can be used to judge whether to update based on the angle difference, thereby controlling the frequency of beamforming control updates.

#### 2.2.3. Beamforming Control Module

**Figure 5:**
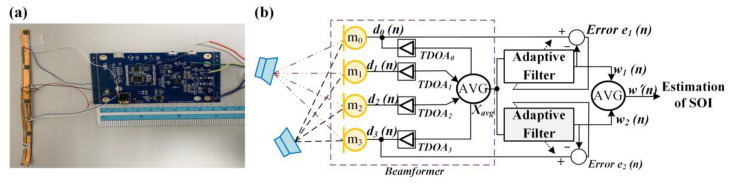
(**a**) Four-array microphone and beamforming module pictures; (**b**) System block diagrams.

In sound source processing, when the distance of the sound source is more than seven wavelengths, it can be called far-field sound, which is different from the spherical wave transmission in the near–field. The far-field is transmitted in the plane wave mode, which determines the direction and intensity of the sensed sound source. Beamforming technology must be used to determine the position and size of the sound source in the far-field. In beamforming technology, the most directional, cost-effective, and structured approach is the adaptive-weight microphone array architecture, which gives different frequency components to different gain and phase adjustments for each microphone output and then adds up all the processed messages. In general, a beamforming system consisting of two microphones can only produce the maximum degradation in any given direction, so the effect will be significantly reduced when the same frequency noise is present at both locations at the same time. In general, two omnidirectional microphone-based beamforming circuits can eliminate one different noise sources. The base calculus for implementing adaptive array beamforming is called Least Mean Square (LMS). When microphone m_0_ collects SOI and noise at the same time, it is assumed to be closer to the noise source below, so it receives almost only noise. Assuming that both m_0_ and m_1_ receive noise from the same source, other than the delivery path being different, the waveform of noise delivery to m_0_ can be estimated by filtering to compensate for the difference caused by the different delivery path, and the SOI can be obtained by subtracting m_0_ from m_1_. The modular and square architecture of the beamforming technology proposed in this study is shown in Figure 5.

The size of the proposed module is about 90 × 59 × 5 mm. The mainboard includes a USB transmission circuit, four micro-electro-mechanical systems (MEMS) microphones, front-end amplification circuitry, microprocessors, a speaker driving circuit, and ear output. At first, the module performs signal acquisition by four far-field array microphones and from targets seen by the users’ eyes. These noises and sounds are amplified and filtered by the preprocess circuit, and the time delay between each microphone is calculated through the TDOA to estimate the direction of the source of the signal. In the beamforming control, we used the Minimum Variance Distortionless Response (MVDR) in adaptive algorithm following the references [31,32]. In addition, CLEAN-SC (Clean based on Source Coherence) was utilized to improve the performance of MVDR. CLEAN-SC is a very effective tool to remove dominant noise sources [32]. Such a beamforming system can truly reflect the signal power of the observation direction at the output, simultaneously provide the beam time sequence for further processing, and obtain a set of noise-resistant and enhanced target sound sources. Finally, signals that enhance the target speech are sent to the speaker driving circuit and played over the ear. The adaptive beamforming filter adopted in the study was used to enhance target sounds of interest and simultaneously suppress uninteresting sounds and ambient noise in feature tags. The purpose of the adaptive beamforming filter was to optimize sound signals in the direction assigned through the weight ratio. In the application of beamforming technology, the adaptive behavior of beamforming could be effectively controlled when the signal gain had not been obtained.

**Figure 6:**
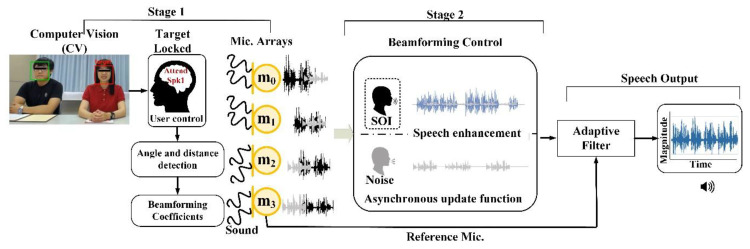
Sound source processing flowchart.

Short-time Fourier transform (STFT) was commonly used to convert the original waveform to a frequency domain feature over a set of Fourier bases from previous processing steps for speech signals. The speech enhancement and enhancement system proposed in this study uses one or more people at its input, as shown in Figure 6. When target SOI, uninteresting human noises, and background noise sounds were entered simultaneously, CV determines the most likely target location of interest and calculates the angle and distance to beamforming control module. In addition, adaptive filter that based on reference microphone analysis was utilized to retain the specific spectrum of speech and reduce the high frequency noise.

## 3. Experimental Design and Results

In order to verify the real-time and noise reduction of the proposed system, a series of experimental tests were performed. During the experiment, noise settings and target speech reception were carried out in the general environment in different azimuths so as to observe the noise reduction effect of the system through four array microphones and circuits under different noise environment settings. In addition, in social conversations with multiple people, the brain can only process one target voice at a time, but it can achieve the purpose of multi-person communication by fast switching [33]. In the system of machine hearing, most of them also focus on a single voice target as the source of speech recognition. Therefore, the device design proposed in this study only focused on a single speech of interest at the same time to confirm the feasibility of the method. Three male and one female are involved as subjects in the experiments and the average age is 26. All the experiments were performed 10 times, the speech sound of SOI with acquisition for 180 s. In order to reduce the interference caused by switching, we removed 40 s of the signal from the beginning and end, and only analyzed 100 s of each recording. Furthermore, at least three of ten experiment, the SOI was from female. The experiment is described below.

### 3.1. Speech Separation Experiment in Different Noisy Environments

In order to simulate the real situation, the test environment of this study was in a general laboratory, in which the subjects read specific manuscripts directly in the noise environment. During the same time, we carried out the recording of traditional omnidirectional type and study device. In addition, in order to ensure the consistency of experimental results, the background noise was confirmed to be below 50 dB before each experiment started. All comparison benchmarks were the results obtained by comparing the recording of omnidirectional with the device proposed in this study. The noise and target sound source energy were set based on a commercially available Standard Sound Level Meter (TES, TES-1350A Sound Level Meter, Taipei, Taiwan) with a range of 70–74 dB, respectively. The SNR_p_ defined based on Equation (5).

(5) $${\mathrm{SNR}}_{p}\left(\mathrm{dB}\right)=20\log_{10}\frac{A_{SOI}}{A_{B}}-20\log_{10}\frac{A_{SOI}}{A_{O}}=20\log_{10}\frac{A_{O}}{A_{B}}$$

where, $A_{SOI}$ denotes amplitude of SOI, and $A_{B}$ denotes the reception of signal amplitude in this study; $A_{O}$ denotes the amplitude of omnidirectional recording sound signal. The detailed experimental structure and results are described below.

#### 3.1.1. Performance Experiment for Target Speech Separation in Noisy Environments

**Figure 7:**
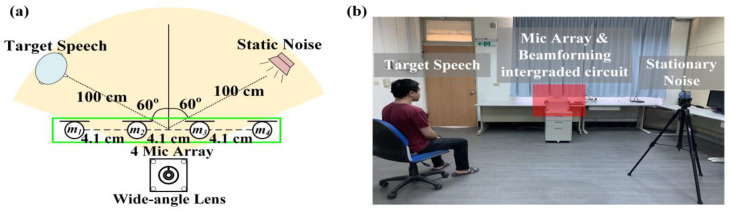
(**a**) Simulation experiment environment schematics; (**b**) Stationary noise and target sound source configuration scenario.

**Figure 8:**
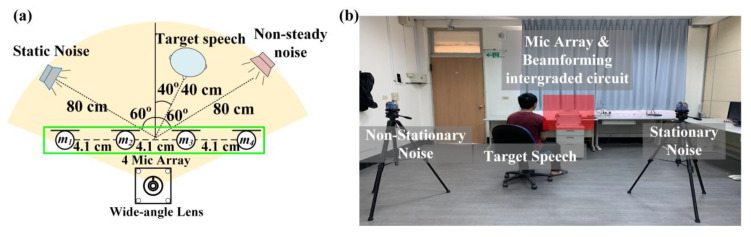
(**a**) Simulation experiment environment schematics; (**b**) Configuration scenarios of non-stationary noise and target sound source.

The experimental architecture is shown in Figure 7 and Figure 8. Four arrays of microphones with a spacing of 4.1 cm were proposed in this study. At first, that target sound source and the noise source were set to be 100 cm away from the center of the system. The target sound source angle and the noise source angle of the experiment shown in Figure 7 were both 60°. This experiment aimed to confirm that the method could effectively identify the target sound and eliminate stable noise interference. The experiment shown in Figure 8 adjusted the target sound source to be close to the noise sources. In this study, we had two kinds of noise sounds. One is stationary noise from Standard Gaussian white noise, the other one is 16 kHz non-stationary noise and both are from Duan et al. in 2012 [34,35]. Furthermore, the speech from non-interest subject was also defined as the non-stationary noise in the experiment. Overall, the interference condition of the experiment in Figure 8 was larger than that in Figure 7, with the aim of confirming that the method could effectively eliminate noise and enhance target speech in the presence of the various noise environment.

**Figure 9:**
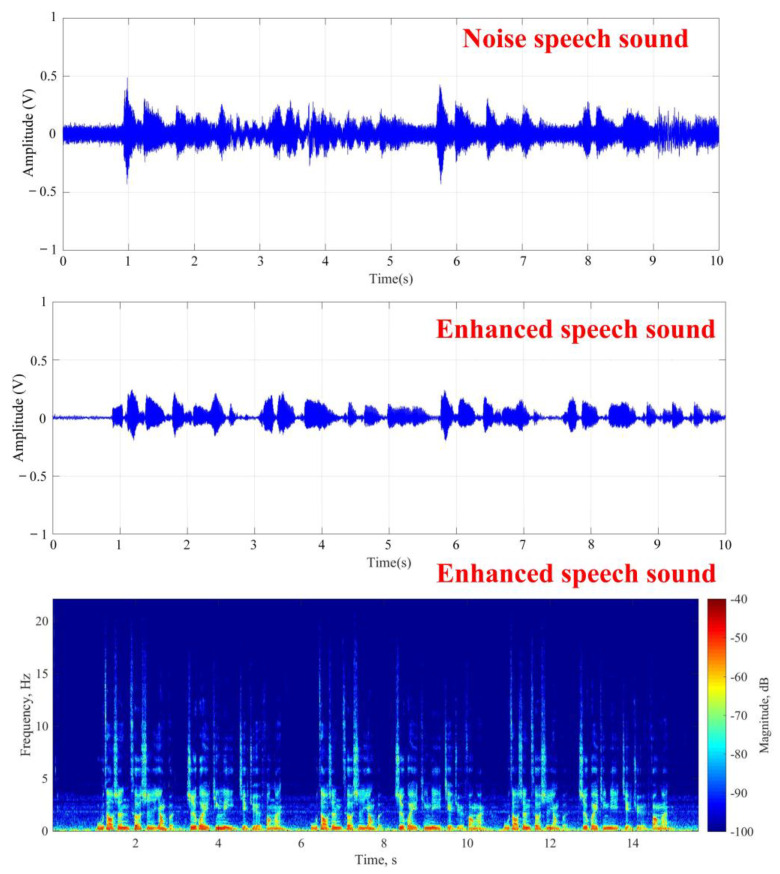
Results of target speech enhancement in white noise environment.

After the start of experiment shown in Figure 7, the target subject would read a text as the target speech and the frontal speaker generated the white noise simultaneously. As to the result of the stationary noise elimination, Figure 9 respectively shows the target speech in the noisy environment, the target speech after noise reduction, and the frequency spectrum of the target speech. The experimental results show that beamforming could effectively suppress the interference of stationary noise sources by forming a fixed angle to enhance reception after the target face features were identified by the system. The average signal-to-noise ratio was 6.1 ± 0.3 dB. The results indicated that the proposed architecture could separate target speech in stationary noise environment.

**Figure 10:**
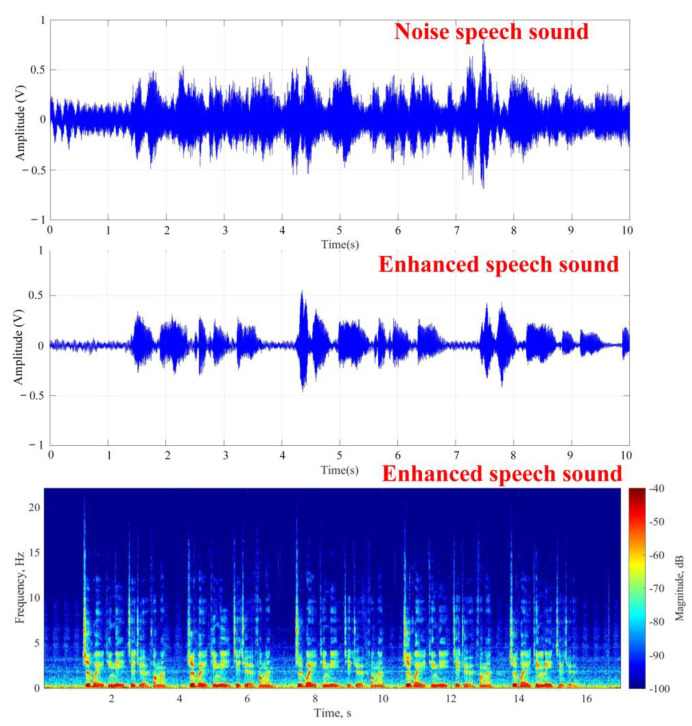
Results of target speech enhancement in various noise environments.

In the stationary noise experiment, the non-stationary noise was added to the experimental conditions in Figure 8 and the target speech source was placed close to the noise source from the speaker to simulate ordinary daily life scenarios. As to the result, Figure 10 respectively shows the target speech in the noisy environment, the target speech after noise reduction, and the frequency spectrum of the target speech, with an average of 5.2 ± 0.4 dB. Although the effect was not as good as that shown in Figure 9, according to the results, the noise could still be effectively eliminated and the target speech signal could be enhanced. Therefore, it could be verified that the structure of the image recognition and beamforming technology could effectively enhance target speech and suppress noise in environments with multiple human voices and stationary noises in daily life.

#### 3.1.2. Moving Target Speech Separation Experiment in Various Noise Environments

**Figure 11:**
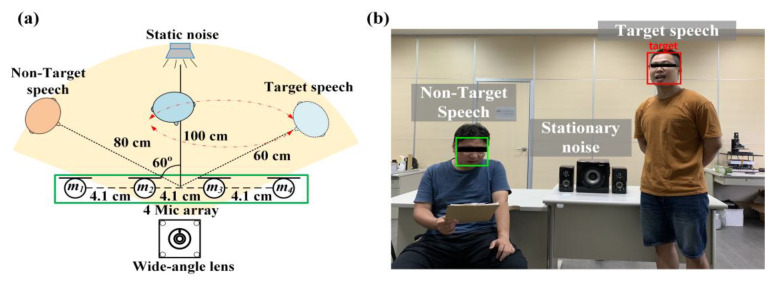
(**a**) Simulation experiment environment schematics; (**b**) Actual multiple noises and moving target.

**Figure 12:**
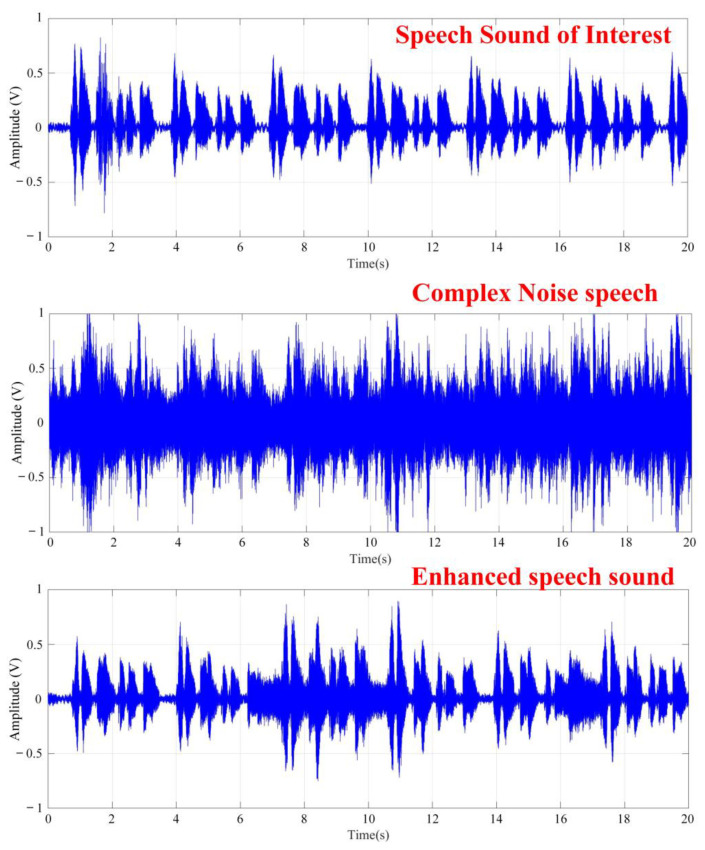
Speech sounds in moving interest targets, non-target speech sounds and filtered speech sounds in various noise environments.

In this section, for both stationary noise and non-stationary human sound environments, the system performed an experiment on the real-time speech enhancement effect of a moving target sound source, as shown in Figure 11. In this experiment, two subjects were used as moving target sound sources and non-target noise sources, respectively, and stationary noise was maintained. Target subject will move from 140° to 50° and then returned to 140° in 180 s, moving back and forth once. (the distance between the camera and subject is 60 cm). Figure 12 shows the moving target speech and the noisy filtered speech sound. The results indicated that the beamforming was continuously tracked and enhanced as the method identified the target sound source. When the SOI and the stationary noise angle were close, the SNR_p_ did not affect speech comprehension, although the noise could not be suppressed completely. Overall, the proposed method could effectively reduce noise by 6.1 ± 0.3 dB in a stationary noise environment and 5.2 ± 0.4 dB in a mixed stationary and non-stationary noise environment. The performance of SNR_p_ was helpful for speech separation.

## 4. Discussion and Conclusions

According to the results of previous studies [36,37] the efficiency of speech enhancement could be improved by beamforming technology but real-time speech could not be significantly enhanced. In those methods, noise speech was divided into different frequency bands, and the gain corresponding to the specific frequency band was adjusted to enhance speech intelligibility according to the use environment and the patients’ degree of hearing loss. However, when there were more than three non-stationary human voices in the formed beam, the level of speech comprehension for a particular target was significantly reduced. Although methods based on deep and recursive neural networks can effectively separate and identify voices, there was a lack of discussion on speech processing in real-time [38,39]. It is difficult to correctly identify the source location of SOI in the information retrieved through beamforming in Stage 1 and perform non-stationary human sound intensification at that time. To improve this problem, this study integrated image processing techniques to quickly and effectively identify SOI with known targets, resulting in improved TDOA estimates. In this study, four far-field MEMS microphones and a beamforming system were used to efficiently collect target speech and suppress noise. According to the experimental results, although the response speed of the proposed system was 40 ms, the response time for actual sound reached 7 ms and the overall recognition rate reached 80% by using asynchronous update technology. Later, in the moving interest target speech enhancement experiment, based on the reception and noise reduction results of four arrays of microphones, after the system identified the target through image analysis, as the target sound source moved randomly, the noise reduction effect would be affected by background noise at 6~12 s. As the SOI moved at random with the same angle as the background noise, it led to higher noise energy of the incoming beam, and this noise could not be suppressed completely at that time. However, in daily use, the system proposed in this study could improve speech by 4~6 dB and provide a better speech comprehension effect for users [3,4]. In addition, in order to realize the real-time system for non-stationary human speech separation, the asynchronous update function was used in CV and beamforming control. After capturing the SOI target angle with the highest probability in the initial state, the beam angle was re-computed and updated every 30–50 ms, which does not affect the real-time speech processing and voice output.

In summary, this study is able to successfully develop a system that can be used in a various noise environment. Although currently only a single target can be focused on at the same time for speech separation, due to the processing time of this method was less than 10 ms, we believe that the method of fast switching could achieve the multi-targets fast switching and focusing. The system can effectively capture multi-person targets and guess interesting speech targets based on distance and mouth movements, thus allowing users to receive enhanced target speech signals and suppress ambient noise. Finally, to facilitate daily use, flexible printed circuit using four MEMS microphones can be integrated with the lens frame. Miniaturized wide-angle cam and TDOA image processing technology can be used to achieve smart lens applications. The proposed method has high potential to be applied in the real-time speech processing system, such as the auxiliary listening device to understand target speech much more clearly in various noise environment. Moreover, it also can be applied in the machine language processing system, like for intelligent personal assistant for the high-quality speech separation identification results [40,41]. Although based on the design of this study, the target speaker needs to face the camera for locating and direction detecting, it may be limited in some situations. Therefore, we think the proposed method was suitable for a speech enhancement and separation function of auxiliary listening device when face-to-face communication. For the machine hearing application, using different angle camera or tracking camera could obtain full sphere panoramic video image without dead angle to overcome this advantage.
